# 

**Published:** 1896-11-07

**Authors:** 


### EARLY EDITION.

**Notices of Births, Deaths, and Marriages** are inserted in this column at a charge of 2s. 6d. for an announcement not exceeding four lines.

### NOTICE TO CORRESPONDENTS.

All MS., Letters, Books for Review, and other matters intended for the Editor should be addressed The Editor, "The Hospital," The Hospital Building, 28 & 29, Southampton Street, Strand, W.C.

The Editor cannot undertake to return rejected MS., even when accompanied by stamped directed envelope.

**Notice to Advertisers and Subscribers.**

All Advertisements, Orders for Copies of The Hospital, and Business Communications should be addressed to **THE MANAGER, (Not to The Eitdor),** The Hospital, The Hospital Building, 28 & 29, Southampton Street, Strand, London, W.C.

The Cost of Subscription, post free, is as follows:—Per week, 2½d.; per quarter, 2s. 8d.; per half-year, 5s. 3d.; per annum, 10s. 6d. Foreign:—Per annum, 15s. 2d.; payable, in all cases, in advance.

**IMPORTANT NOTICE.**

The EDITORIAL and PUBLISHING OFFICES of "THE HOSPITAL" are now TRANSFERRED to their new premises, THE HOSPITAL BUILDING, 28 & 29, SOUTHAMPTON STREET, STRAND, LONDON, W.C.

**NOTICE.—Vol. XX. of "The Hospital" (April to September, 1896), in handsome cloth binding, is now on sale at the Office of this Journal, price 7s. 6d. Cases for binding the Numbers contained in this Volume 1s. 6d. Index to Vol. XX., 2½d., post free.**
**The Monthly Part for November will be ready on November 2nd, Price 6d., by post 8d.**

### Scraps and Gleanings.

The expenses of the Hull Hospital Sunday Fund last year amounted to just over 5 per cent. of the total collection.

In twelve years the in-patients treated at the County and City of Cork Hospital for Women and Children have increased from 193 to 470, and the daily average number from a little over 19 to 54.

Something like 25 per cent. of the patients discharged each year from the St. George's Hospital are admitted to the Atkinson Morley's Convalescent Hospital at Wimbledon, established in connection with that hospital.

The report of the Metropolitan Hospital contains a capital idea. Illustrations of various wards are given, each bearing an intimation that donors of a certain sum can endow and name a bed in the particular ward they are looking at.

It is proposed to hold a concert this winter in aid of the Metropolitan Hospital, Kingsland Road. Help in selling tickets and in other ways will be much appreciated. All communications should be addressed to Mr. L. Van Boolen, 26, Sandringham Road, Dalston, N.E.

On January 1st, 1895, 623 patients remained under treatment in the three Madras Lunatic Asylums. Two hundred and twenty-one cases were admitted during the twelve months following, making the total number of cases treated during 1895 844. The total expenditure amounted to Rs. 79,228.

The total number of in-patients at the Roosevelt Hospital, New York, in 1895 (3,264) was 380 more than the previous highest record in the history of the institution; while the number of accidents (5,485) was 466 above the number treated in any previous year. The net cost per in-patient per day was $1·87, as against $1·74 in 1894.

An interesting fact in connection with hospital appeals is that in response to a special appeal sent out by a West of England general hospital only 100 replies were received, although 2,000 specially-selected names were sent to. Of these 100 donors five contributed £1,000 between them, and the remaining 95 a sum of £262.

During 1895 1,360 patients were treated in the wards of the hospital of the University of Pennsylvania, at Philadelphia, of whom 775 occupied pay beds, 353 free beds, 80 endowed beds, and the remainder were accident cases. 9,117 patients were treated in the dispensaries attached to the hospital, 1,285 in the receiving ward, and 64 in their own homes.

We are glad to see from the last report of the Roosevelt Hospital, New York, a note that "the lack of uniform method in reporting hospital statistics" in the United States of America "has received some consideration by the local association of hospital superintendents at their meetings throughout the year." Our pleasure is, however, somewhat marred by the fact that "a comprehensive scheme has not yet been perfected."

The New York Hospital, which includes the hospital proper, a department for the insane at White Plains, and a house of relief in New York, during the year 1895 treated in all departments 39,318 patients, of whom 64 per cent. received free treatment. These patients included 5,320 in and 9,803 out patients at the New York Hospital, 2,704 in and 21,057 out patients at the House of Relief, and 434 insane patients at the Hospital for the Insane.

### Books Received.

R. Bernard.

"A B C Medical Guide."

Swan Sonnenschein and Co.

"Premature Burial." By William Tebb, F.R.G.S., and Colonel Edward Perry Vollum, M.D.

"Problems of Biology." By George Sandeman, M.A.

J. and A. Churchill.

"Lectures on Medicine to Nurses." By Herbert Cuff, M.D., F.R.C.S.

A. D. Innes and Co.

"The Green Garland." By Frances E. Crompton.

Institut International de Bibliographie Scientifique (Paris).

"Traitment des Suppurations Pelviennes." Par Eugène Doyen.

South Counties Press (Lewes).

"The Increase in General Paralysis in England and Wales." By R S. Stewart, M.D., D.P.H.

### The Student of Medicine.

### APPOINTMENTS.

P. E. Adams, M.R.C.S., L.R.C.P., appointed House Surgeon to the Dorset County Hospital. R. H. Ashwin, L.R.C.P.Lond., M.R.C.S.Eng., appointed Assistant House Surgeon to the County Hospital, York. K. D. B. Dobbs, L.R.C.P, L.R.C.S.I., appointed Medical Officer for the Tutbury District of the Burton-on-Trent Union. P. H. Dunn, L.R.C.P.Lond., M.R C.S.Eng., appointed Medical Officer for the Fourth District of the Hitchin Union. C. T. Farsons, M.D.Lond., appointed Senior Assistant Medical Superintendent to the Fulham Union Infirmary. Mr. Gardner, appointed Honorary Surgeon to the Torbay Hospital. R. H. H. Hayden, L.R.C.P. and S.I., appointed District Surgeon of Sutherland, Cape Colony. Dr. G. H. Jenkins, appointed Medical Officer for the Usk District of the Pontypool Union. G. W. Johnstone, L.R C.P.Edin., L.F.P.S. Glas., appointed Medical Officer for the Upholland District of the Wigton Union. F. P. Jones, M.R C.S.Eng., L.R.C.P.Lond., appointed House Physician to the Swansea General and Eye Hospital. J. H. Morris, M.R.C.S.Eng., L.S.A., appointed Medical Officer for the Second District of the Salford Union. L. F. Nash, L.R.C.P. & S.Edin., appointed Medical Officer to the Elizabeth Fry Refuge, Mare Street, Hackney. R. P. O'Gorman, L.R.C.P., L.R.C.S.Irel., appointed Medical Officer for the Fourth District of the Salford Union. F. E. De Beeho Pim, L.R.C.P., L.R.C.S.Irel., appointed Medical Officer for the Pendle District of the Burnley Union. C. E Potter, M.B., C.M.Edin., appointed Medical Officer for the Hartingdon District of the Ashbourne Union. E. L. K. Pringle, M.B.Edin., appointed Honorary Surgeon to the Bridgwater Infirmary. Mr. M. W. Renton, appointed Medical Officer for the Luddington District of the Goole Union. F. S. Stanwell, M.B., C.M.Edin., appointed Assistant Medical Officer to the East Riding Asylum, Beverley. F. T. Thistle, L.R.C.P.Lond., M.R.C.S.Eng., appointed Honorary Physician to the Torbay Hospital. F. M. Turner, M.D.Camb., appointed Medical Superintendent to the South-Eastern Fever Hospital. Dr. J. G. Williamson, appointed Medical Officer for the First District of the East Ashford Union. W. A. Winship, M R.C.S.Eng., L.S.A., appointed Medical Officer for the Second Division of the Penrith District of the Penrith Union. Dr. Woodforde, appointed Medical Officer of Health to the Easthampstead District Council. P. T. Finn L.R.C.P., L.R.C.S.Edin., L.F.P.S.Glas., appointed Assistant Medical Officer to the Isle of Wight County Council. Mr. Langworthy, appointed Medical Officer for the Fourth District of the Plympton Union. Mr. Potter, appointed Medical Officer for the Hartington District of the Ashbourne Union. T. C. Hayes, M.D., F.R.C.P., appointed Professor of Practical Obstetrics at King's College.

### UNIVERSITIES AND COLLEGES.

### Society of Apothecaries of London.

Pass List (October, 1896).

The following candidates passed in:

Surgery.—G. F. M. Clarke, Charing Cross Hospital; T. S. Collin, Manchester; G. W. Dutton, Middlesex Hospital; F. R. M. Heggs, Birmingham; W. J. Henson, Guy's Hospital; E. L. C. Muspratt, King's College Hospital; H. S. Oliver, Charing Cross Hospital.

Medicine, Forensic Medicine, and Midwifery.—G. E. Brooke, Cambridge and London; A. V. Moreton, Manchester; F. H. Wilkinson, Liverpool; J. B. Wall, St. Mary's Hospital.

Medicine and Forensic Medicine.—T. H. Guillaume, Dublin and Charing Cross Hospital.

Medicine and Midwifery.—A. Avent, St. George's Hospital; G. S. Thompson, St. George's Hospital.

Medicine.—J. Gott, King's College Hospital; A. P. Square, Middlesex Hospital.

Forensic Medicine and Midwifery.—R. S. Thompson, Belfast.

Midwifery.—A. J. Hull, Guy's Hospital; A. C. McLean, King's College Hospital.

The diploma of the Society was granted to the following candidates: Messrs. Collin, Heggs, McLean, Moreton, Oliver, and Wall.

### St. George's Hospital Medical School.

The annual distribution of prizes and certificates was held in the Board-room of St. George's Hospital on Thursday, the 29th inst., at Three p.m. Dr. Wadham, formerly dean of the school, presided, and made the distribution. The following prizes and certificates were awarded: Mr. Adrian Caddy, an entrance scholarship in arts of £145; Mr. Hubert St. George Goldsmith, an entrance scholarship in arts of £50; Mr. John Henry Tallent, B.A., and Mr. Beaumont Percival, B.A., both of St. John's College, Cambridge, entrance scholarships in science of £85 each; Mr. Horace Middleton Vernon, B.A., the William Brown £40 exhibition, the Brackenbury prize in medicine, and the Brodie prize in surgery; Mr. Charles Ralph Keyser, the Brackenbury prize in surgery; Mr. Henry Goodridge Deller, B.A., the Webb prize in bacteriology; Mr. Harold Merriman Cooper, the Treasurer's prize; Mr. John Howell Evans, B.A., the Acland prize; Mr. Henry George Drake Brockman, the Henry Charles Johnson prize in anatomy, the second year's general proficiency prize, and a certificate of honour in physiology; Mr. Lawrence Pick, the Pollock prize in physiology; Mr. Henry Neville Coltart, the Sir Charles Clarke's prize; Mr. Wentworth Francis Tyndale, the third year's general proficiency prize; Mr. Harold Robert Dacre Spitta, the first year's general proficiency prize; certificates of honour in anatomy, Mr. Frederick Paul and Mr. Arthur S. Morley; certificate of proficiency (second year), Mr. Miller Neatby, M.A.; certificate of proficiency (first year), Mr. Lidbrook Frank Cope.

